# Climate forcing and desert malaria: the effect of irrigation

**DOI:** 10.1186/1475-2875-10-190

**Published:** 2011-07-14

**Authors:** Andres Baeza, Menno J Bouma, Andy P Dobson, Ramesh Dhiman, Harish C Srivastava, Mercedes Pascual

**Affiliations:** 1 Deparment of Ecology and Evolutionary Biology University of Michigan, Ann Arbor, MI, USA; 2 London School of Hygiene and Tropical Medicine. University of London, London, UK; 3 Department of Ecology and Evolutionary Biology, Princeton University, Princeton, NY, USA; 4 National Institute of Malaria Research (ICMR), Delhi, India; 5 National Institute of Malaria Research, (ICMR), Field Unit, Civil Hospital, Nadiad, Gujarat, India; 6 Howard Hughes Medical Institute, Chevy Chase, MD 20815-6789, USA

## Abstract

**Background:**

Rainfall variability and associated remote sensing indices for vegetation are central to the development of early warning systems for epidemic malaria in arid regions. The considerable change in land-use practices resulting from increasing irrigation in recent decades raises important questions on concomitant change in malaria dynamics and its coupling to climate forcing. Here, the consequences of irrigation level for malaria epidemics are addressed with extensive time series data for confirmed *Plasmodium falciparum *monthly cases, spanning over two decades for five districts in north-west India. The work specifically focuses on the response of malaria epidemics to rainfall forcing and how this response is affected by increasing irrigation.

**Methods and Findings:**

Remote sensing data for the Normalized Difference Vegetation Index (NDVI) are used as an integrated measure of rainfall to examine correlation maps within the districts and at regional scales. The analyses specifically address whether irrigation has decreased the coupling between malaria incidence and climate variability, and whether this reflects (1) a breakdown of NDVI as a useful indicator of risk, (2) a weakening of rainfall forcing and a concomitant decrease in epidemic risk, or (3) an increase in the control of malaria transmission. The predictive power of NDVI is compared against that of rainfall, using simple linear models and wavelet analysis to study the association of NDVI and malaria variability in the time and in the frequency domain respectively.

**Conclusions:**

The results show that irrigation dampens the influence of climate forcing on the magnitude and frequency of malaria epidemics and, therefore, reduces their predictability. At low irrigation levels, this decoupling reflects a breakdown of local but not regional NDVI as an indicator of rainfall forcing. At higher levels of irrigation, the weakened role of climate variability may be compounded by increased levels of control; nevertheless this leads to no significant decrease in the actual risk of disease. This implies that irrigation can lead to more endemic conditions for malaria, creating the potential for unexpectedly large epidemics in response to excess rainfall if these climatic events coincide with a relaxation of control over time. The implications of our findings for control policies of epidemic malaria in arid regions are discussed.

## Background

The response of epidemic malaria to large-scale change in land-use practices related to irrigation and agriculture in arid regions remains poorly understood [1]. In the last three decades, for example, the expansion of a large network of irrigation canals has supplied an important source of freshwater for agriculture in many arid regions of India; in so doing, it has also contributed to the economic development of these regions. More generally, change in irrigation schemes, and associated agricultural practices, are considered among the potential drivers underlying malaria's increasing global burden [2], but their consequences remain poorly understood given the complexity of their effects on transmission via human wealth and vector ecology. In particular, it is not clear how irrigation is modifying the coupling of epidemic malaria to rainfall variability in arid regions.

The population dynamics of malaria at the edge of its distribution, in either deserts or highlands, where rainfall and temperature respectively limit transmission, are characterized by strong seasonality and significant variation in the size of outbreaks from year to year [e.g. 3-5]. In these regions the role of climate forcing is potentially central to the prediction of inter-annual variability of epidemics. The high variability in the number of cases between years challenges public health efforts, as severe intermittent epidemics can strain medical facilities.

In the north-west of India, there has been a long-standing interest in the development of early-warning systems based on rainfall [6,7] and economic conditions [8]; this has regained significance in the last decades following the failed eradication attempts in the 1960's and 70's. Epidemics have re-emerged in the desert states of Rajasthan and Gujarat, and have once again motivated interest in climate forcing [9,10] and its interplay with socio-economic factors. An increment in burden has been attributed to the extension of the canal network that provides water for regional agriculture [11].

Despite the potential of remote sensing for the generation of early-warning systems [12], efforts have been largely focused on defining malaria's spatial niche and seasonal timing [13,14] rather than on predicting the seasonal burden in areas of unstable malaria (but see [15] for dry regions of Eritrea). Because vegetation can be used as a proxy for the amount of water in the ground and, therefore, for the humidity of the environment, the Normalized Difference Vegetation Index (NDVI) provides a spatially-explicit link between rainfall and malaria from local to regional scales. Thus NDVI patterns are especially relevant for investigating the relationship between epidemic events and regional climatic drivers in desert regions [16,17]. However, any vegetation index will be susceptible to landscape changes due to irrigation and agricultural practices. The extensive time series of malaria cases in desert and semi-desert districts of Rajasthan and Gujarat provide an opportunity to examine how NDVI-malaria associations change across regions that represent a gradient in levels of irrigation.

This paper addresses how the level of irrigation modifies the dynamics of malaria across such a gradient and how irrigation affects the predictability of epidemics based on climatic variables. It further investigates whether a decoupling of rainfall forcing and malaria epidemics with increased levels of irrigation reflects (1) a real reduction in the risk of transmission, (2) more effective control efforts but no reduction in disease risk, or (3) a breakdown of NDVI as a sensitive indicator of rainfall's inter-annual variability. These different mechanisms have very different implications not just for prediction but for the possibility of unexpected epidemics when control efforts fail. Statistical analyses are used to address these different hypotheses, and the findings are discussed in light of a simple dynamical model of mosquito abundance and irrigation. The data provide additional empirical evidence for the possibility of surprises in years when intervention and monsoon rains vary simultaneously but in opposite directions. This suggests that control policies based on residual insecticide spraying, whose planning is "reactive" to disease levels in the previous season, need to be modified given the known consequences of past failures and relaxation of control in irrigated areas. The implications of these findings for forecasting and control policies in other arid regions with epidemic malaria are also discussed.

## Methods

### Malaria and remote sensing data

#### Epidemiological data

**Figure 1:**
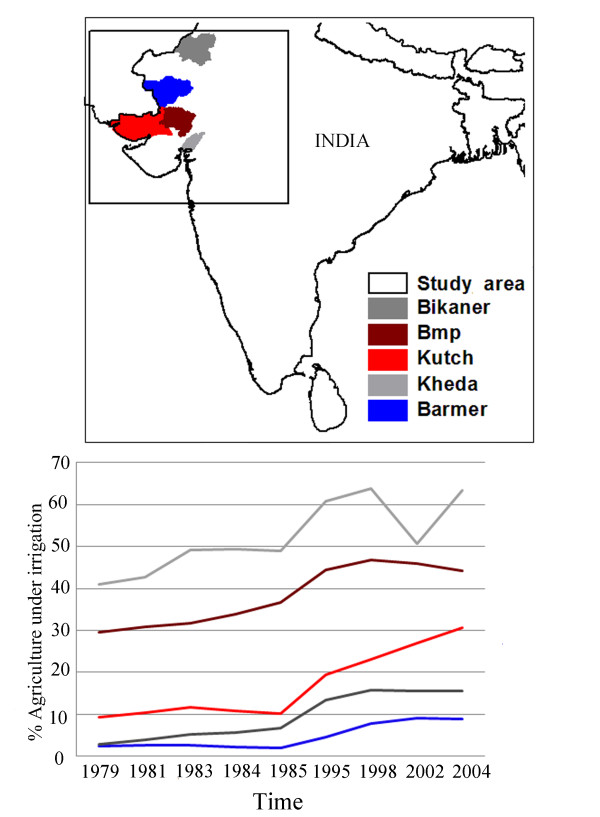
**Study area in north-west India and the level of irrigation of each district**. Each line represents a time series of the percentage of agricultural land under some source of irrigation (source: district statistical books, Gujarat and Rajasthan).

The epidemiological data consists of time series of monthly confirmed cases of *Plasmodium falciparum *from five districts in north-west India (Figure 1): Bikaner and Barmer in Rajasthan, and Kutch, Kheda, and the combined area of Banaskantha, Mehsana, and Patan (hereafter, BMP) in Gujarat state. The data from BMP was combined into one dataset to circumvent problems related to modifications of boundaries over the time of this study, and the resulting separation into different districts of what was originally a single administrative unit at the beginning of the data collection (1976). For Bikaner, BMP and Kheda, the data represent 30 years of observations (1976-2009), and for Barmer, 24 years (1985-2009 with a gap between 2004 and 2006). For each time series the monthly cases were divided by the total yearly human population of the respective district, and all analyses relied on this normalized incidence data (See Additional file 1, Figure S1). The blood samples were collected by passive surveillance of patients that visited their local health facility, and by active surveillance of patients with fevers in house-to-house visits. These data were obtained from the offices of the Joint Director, Vector Borne Diseases, Commissionaire of Health Rajasthan and Gujarat.

Information on the level of irrigation was extracted from statistical abstract books from the states of Gujarat and Rajasthan between 1979 and 2006. For each district the percentage of agriculture under some type of irrigation was computed by dividing the number hectares of agriculture by the number of hectares of land under irrigation (Figure 1). These quantities were then ranked by district based on these irrigation levels. The resulting ranking remains unchanged for the whole time period covered by this study.

#### The Normalized Difference Vegetation Index

NDVI is defined as the difference in radiation reflected by any surface in two bands of the energy spectrum - the infrared and the red band. From this index, which ranges from -1 to 1, it is possible to discriminate between radiation reflected by vegetation and other surfaces. Values greater than 0.2 quantify vegetation greenness from different sources and/or from different seasons. The NDVI data utilized in this analysis was obtained from two sources that cover respectively two different time periods. The first one is the Global Inventory Modeling and Mapping Studies (GIMMS) [18]. The original source of this product is a combination of observations made by different NOAA missions carrying the Advanced Very High Resolution Radiometer (AVHRR). This dataset has a temporal extension ranging from 1982 to 2006 on a bi-monthly basis, and a spatial extension covering the entire globe at a resolution of 8 km. The data have been corrected to avoid distortions and to show only positive values [18]. A window area that includes the five districts from which we extracted the vegetation information (19.18°N - 29.15°N and 66.98°E - 78.55°E; Figure 1) was defined for the years with epidemiological information up to 2006. The second source corresponds to the NDVI product from the Moderate Imaging Spectroradiometer (MODIS) in the TERRA satellite. The data are distributed by the Land Processes Distributed Active Archive Center [19] located at the U.S. Geological Survey (USGS) Earth Resources Observation and Science (EROS) Center. Specifically, all the analyses with MODIS rely on the monthly average product at 1 km resolution for the month of September from 2000 to 2009.

The statistical analyses focused on the longer NDVI dataset from NOAA (referred to hereafter as NDVI) and corroborated that similar results for the spatial correlation maps (see below) were obtained with the shorter but more recent MODIS NDVI.

#### Rainfall data

Monthly accumulated rainfall records were acquired to compare predictability based on rainfall to that based on NDVI. The data, which span 20 years, were obtained from one local station within each district and supplied by the Indian Meteorological Department in Pune (India).

### Statistical and numerical analysis

#### The signature of NDVI

Irrigation increases water available for agriculture, making yearly multi-crop rotations possible. The differences in water supply and multi-crop rotations, therefore, should be reflected in the amount of vegetation and in the signature of the NDVI time series. In highly irrigated areas we should observe less inter-annual variability than in non-irrigated areas. At the same time, the seasonality of NDVI should also vary across districts as different peak times can result from different crop seasons supported by irrigation. Here, both the coefficient of variation of NDVI for each grid-point (pixel) in the study area and the month at which NDVI shows the largest average value (peak) were calculated as a way to quantify the regularity and seasonality over time for each location in the study area. The coefficient of variation (CV) is a dimensionless measure of the variability of a quantity with respect to its mean value. Therefore, CV allows us to compare different districts independently from their mean value.

#### Correlation maps

Since malaria time series exhibit strong seasonality with a peak of cases between October and December (See Additional file 2: Figure S2), the association between malaria and NDVI was examined for the monthly cases of each of the months of the epidemic season (October, November and December) and NDVI at a given preceding month for each year. Spearman rank correlation was used as a non-parametric measure of association between incidence in a given month (say October) and NDVI (say in September). A map of the NDVI/malaria correlation was obtained by computing this correlation repeatedly for each 8 × 8 km grid point in the study area. These maps provide one way to discern the hypothesis that rainfall, as a regional phenomena, no longer acts as a driver from the alternative that it continues to do so, but it is poorly reflected in the local NDVI. For this purpose, a large regional box overlapping the Thar desert and the area within the boundaries of a given district are relevant. The former provides information on NDVI at a large regional level (and, therefore, climate), and the latter corresponds to a more local level under the influence of district land-use and irrigation practices. The resulting correlation map shows the correlation coefficient only for those grid points in which its value is statistically significant (p < 0.05 for NOAA and p < 0.1 for MODIS). These maps were obtained for both NDVI products.

In addition, time series were constructed for each district for the average NDVI over an area of approximately 576 km2, selected because it exhibited the highest rank correlation within the given district. These time series were then used to fit parametric linear regression models of malaria cases for a given epidemic month as a function of NDVI in the preceding months. Since rainfall has been of interest as a predictor variable in early-warning systems for desert malaria, the proportion of the variance explained by NDVI was compared here to that explained by accumulated rainfall, for every month preceding the epidemic season. This allowed a comparison of the predictive skill of NDVI to that of rainfall, and an examination of the respective delays between NDVI, rainfall, and epidemics. Rainfall was accumulated over the monsoon months based on previous results indicating the usefulness of the resulting quantity in malaria transmission models for this region [20].

#### Wavelet analysis

In order to further investigate the relationship between NDVI time series and malaria, the spectral signature of both times series was obtained using wavelet analysis. Wavelet analysis is particularly well suited for studying the dominant periodicities of epidemiological time series because of the non-stationary nature of disease dynamics [21-23]. By contrast to the standard Fourier power spectrum, which provides a global analysis (over time) of the dominant frequencies of a time series, the wavelet spectrum is local in time, providing the additional information of when a specific frequency is present (for a detailed explanation of the method and the interpretation of the results see [21,22] and [23] for an epidemiological application). Here the implementation developed by Cazelles and collaborators [21] was used which includes the assessment of statistical significance based on bootstraps methods. For each district the wavelet power spectrum was calculated for both the NDVI and the malaria time series.

#### Model and simulations

In order to interpret and discuss the results of the statistical analyses, a simple model of malaria risk was also developed that encapsulates basic elements of the relationship between rainfall, irrigated agriculture and mosquito population abundance (See Additional file 3: A simple model of mosquito population dynamics, rainfall and irrigation). The model consists of a set of differential equations describing how rainfall water is allocated to different compartments of the landscape (See Additional file 4; Figure S4). Simulations of the model are used to examine the consequences of increasing irrigated area for (1) the seasonal and inter-annual correlation between rainfall and mosquito abundance and (2) disease risk measured in terms of mosquito abundance.

## Results

The analyses of the variability and seasonality of the spatially explicit NDVI time series show that NDVI inside BMP and Kheda have a very low coefficient of variation (CV) for a large fraction of grid points, and that the average peak of NDVI occurred more often in the months of January and February (reflecting irrigation for the winter crops). By contrast Barmer and Bikaner both show a higher CV for most locations, and the peak vegetation months fall after the monsoon rains and preceding the epidemic season (in September and October). Thus, the temporal dynamics of NDVI differ across the districts and are altered in the presence of irrigation and associated agriculture (See Additional file 5: Figures S5).

**Figure 2:**
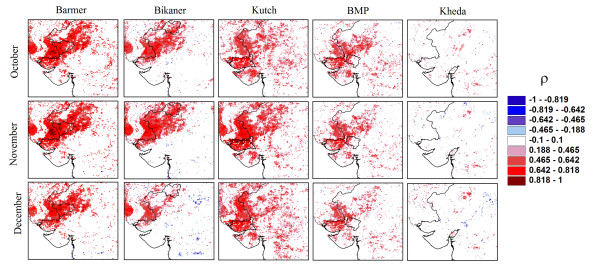
**Correlation maps**. Spearman rank correlation (ρ) between malaria incidence from October to December and September NDVI, for each location (8 × 8 km grid point) of the study area. Each location (pixel) then represents the correlation in time between NDVI at this location and malaria incidence from a specific district. This boundary of this district is indicated inside each map. A high spatial correlation is observed over a large regional area (including the Thar desert), especially for the driest and weakly irrigated districts.

**Figure 3:**
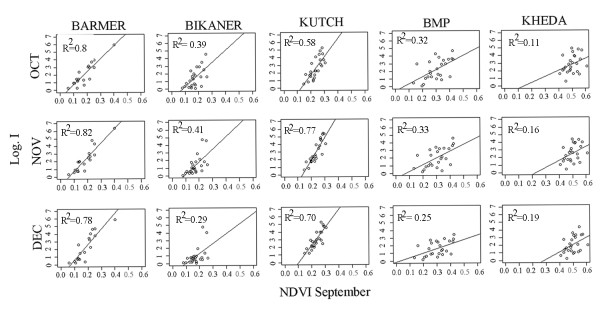
**Linear regression plots**. September NDVI is the predictor of malaria incidence in October, November and December.

Spatial correlation maps (Figure 2) show that the prevalence of malaria in Barmer and Bikaner, the two districts with the lowest irrigation values, has a strong and significant positive association with September's NDVI for a large region including parts of the Thar Desert. Thus, high values of NDVI in the arid zone precede the observation of an important portion of cases in the consecutive months. In the adjacent region with higher levels of irrigation, BMP, only a small part of the district shows a positive and significant correlation with NDVI; although an association can still be observed with regional NDVI from areas outside the district. This suggests that NDVI has weakened as an indicator of rainfall variability but that this variability continues to act to some extent as a driver of malaria at inter-annual time scales. At the highest level of irrigation, Kheda showed a different pattern: no significant correlation with NDVI is present for any location of the study area, inside or outside the district. In this district, rainfall variability appears to no longer act as a driver of epidemic size. Similar results are obtained with the more recent NDVI product, MODIS (See Additional file 6: Figure S6). The parametric linear models show similar results. Barmer, Bikaner and Kutch have a high and significant correlation with a coefficient of determination *R^2 ^*>0.7, whereas for Kheda and BMP, this association weakens considerably (Figure 3).

**Figure 4:**
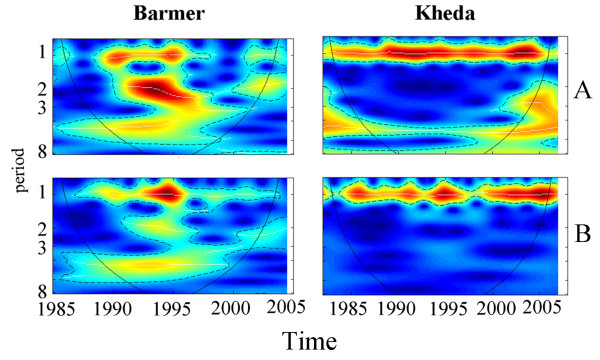
**Wavelet power spectrum for malaria incidence and NDVI**. The wavelet power spectra of the cases (panel A) and NDVI (panel B) are shown for Barmer and Kheda, the two districts at the extremes of the irrigation gradient (for the rest of the districts see Additional file 6, Figure S6). The wavelet spectrum shows the variance (technically the power) for different periods (y-axis) and for different years (x-axis). The scale ranges from blue to red, with red indicating high power at a particular year and period. As irrigation increases, the 1-year period becomes stronger and the 2 and 4-year periods become weaker.

A similar picture emerges from the analysis in the frequency domain. Figure 4 shows the power spectrum for both the malaria and NDVI time series for the two districts corresponding to the extremes of the irrigation spectrum. Results show that for Barmer, malaria and NDVI both show strong and significant power in the 2 and 4-year periods. On the other extreme of the irrigation intensity, the spectrum for Kheda shows that most of the variance is concentrated in the one year period, as expected for seasonal malaria (epidemics), and in the five year period with this multiyear cycle absent in NDVI. NDVI shows some power for period 4, but for a small window of time (between 1992 and 1998). A general pattern observed in the power spectrum of both NDVI and malaria for most all the districts is that as irrigation increases, the time interval during which the seasonal signal (at period one) predominates increases with irrigation (see Additional file 7: Figure S7). In Barmer, for example, the annual signal is absent for considerable extents of time, with the exception of a few years in the early nineties. By contrast, this signal is present in Kheda almost uniformly over time as the dominant scale of variability. Concurrently, the times with variance concentrated around period 2 decrease as the extent of agricultural land under irrigation expands. This is also true for NDVI with the exception of Barmer, which shows less activity than the other districts in this particular frequency. Although the variance of Kheda is concentrated in the seasonal cycle, the average seasonal pattern of malaria incidence in this district also shows a less pronounced seasonality, in the sense that the troughs, in the inter-epidemic months, exhibit higher values than in less irrigated districts (See Additional file 2: Figures S2). At the same time, on average, the seasonal incidence during epidemic months in Kheda is higher (and not lower). Thus, when epidemics occur, disease burden tends to be larger than in the less irrigated districts. This is the case both in earlier and more recent times (See Additional file 1 and Additional file 8: Table S1 for the large outbreaks of 2004).

A comparison between NDVI and rainfall predictability shows that NDVI is a better explanatory variable at a lead time of one month (See Additional file 9). In August, the best predictor month for rainfall, NDVI performed equally well for Barmer, Bikaner, and Kutch. This comparison also shows that for BMP, rainfall is a better predictor than NDVI, and that in Kheda neither rainfall nor NDVI can predict the size of epidemic events, confirming the decoupling of the malaria system from the annual effect of rainfall in the number of cases.

Consistent with the above results, a dynamical model (see Methods and Additional file 3) illustrates that mosquito abundance increases as the area of the landscape under irrigated agriculture increases (See Additional file 10, Figure S10, panel B). However, this increment is only observed for the months in which irrigation is supplied to the agriculture (the driest months) and not during the epidemic season. This would result in less pronounced troughs between epidemics and more sustained transmission. It also follows from this that irrigation cannot modify the inter-annual correlation between rainfall and mosquito abundance in the model (See Additional file 11, Figure S11, panel D). Thus, disease risk remains associated with excess rainfall, in the absence of additional factors such as intervention efforts or the increase in levels of population herd immunity with higher transmission.

## Discussion

The resurgence of malaria in desert and semi-desert areas of Rajasthan and Gujarat over the last three decades once again underscore the potential relevance of an early-warning system based on climate variability. Any new predictive tool must now also consider the considerable changes in land-use patterns that arise from irrigation and agriculture. More generally, the long-term surveillance programmes in India provided an opportunity to address climate forcing in the context of changing irrigation patterns, a problem of relevance to desert malaria in other continents. The results suggest an increasing decoupling of malaria with local and then regional rainfall variability that possibly reflect more effective control measures, rather than a real reduction in potential risk. This has clear implications for control policies as discussed below.

This work specifically examined the relationship between a remote sensing index of vegetation (NDVI) and malaria in districts that differ considerably in levels of irrigation. September NDVI was shown to provide a reliable predictor of malaria prevalence during the epidemic period (October, November and December) both within the district and in the larger region; however, this prediction is strongest in districts where irrigation is low or completely absent. These results provide a bridge between predictions at a somewhat longer lead time, in July and August, based on rainfall and the actual peak of cases in October, November and December. For the non-irrigated districts, September NDVI explained the inter-annual variation in the size of malaria epidemics better than the accumulated rainfall of August and September. The coherence of malaria and NDVI in the frequency domain, with 2 and 4-year cycles, further confirms the importance of the monsoon rains as a driver of malaria's inter-annual cycles in these arid and un-irrigated regions.

In contrast, in the highly irrigated districts, Kheda and in the combined Banaskantha, Mehsana, and Patan (BMP), the association between NDVI and malaria weakens and NDVI provides a poor predictor of the magnitude of an outbreak. For BMP this is only the case for NDVI within the district and not at larger regional scales. This suggests a persistent role of rainfall forcing on malaria transmission, with irrigation and agriculture mainly compromising how well NDVI reflects this variability within the district. For Kheda, the more extreme breakdown of the association with NDVI both inside and outside the district indicates that rainfall no longer determines the size of epidemics from year to year.

Two hypotheses arise as possible explanations of this breakdown: 1) an increase in wealth with irrigation would underlie more effective intervention measures based on residual insecticide spraying, which in turn would prevent epidemics under anomalous and large monsoons. More irrigated, wealthier districts would therefore experience a greater decoupling than poorly or non-irrigated ones. Or 2) the importance of rainfall has diminished due to changes in either the entomological and ecological conditions underlying the vectors' dynamics, or climatic conditions. In particular, multi-crop systems supported by irrigation and rainfall can continually provide the water necessary for a suitable mosquito environment. For example, a study in Pakistan's Punjab region [24], where malaria used to exhibit pronounced monsoon driven epidemics, described how irrigation now provides perennial sites for mosquito breeding, with different vector species breeding at different seasons during the year. On longer time scales, both mechanisms might be at play, with the development of irrigation ultimately resulting in more extensive ecological and socio-economic change and a more permanent reduction of malaria risk [9]. This implies that similar effects may occur in Kheda, which currently exhibits similar epidemic timing, but with a higher disease incidence (when epidemics occur) than in other districts, suggesting that disease risk following anomalous monsoons has not decreased with irrigation, but that intervention measures have prevented its manifestation. Indoor Residual Spray (IRS) in these districts is planned at the village level based on the incidence of malaria in the previous year. All the dwellings in villages whose annual parasite incidence exceeds 2 per thousand population are targeted for spray in the next year and preceding the epidemic season.

Higher levels of irrigation and associated wealth would underlie more effective implementation of planned IRS, and in so doing keep malaria transmission in check at the same time that malaria risk itself would remain responsive to climate variability (and even increase). This creates the possibility of surprises, with unexpected large epidemics when a temporary failure of control coincides with an anomalous monsoon year. An example of this is suggested by data on residual insecticide spraying in Kheda (See Additional file 8; Table S1). In 2004, the number of cases increases dramatically following a series of years of low incidence and low insecticide application. This year also exhibits an excess of rainfall. This large epidemic was then followed by an increase in the level of insecticide spraying and, correspondingly, by low levels of malaria cases, which in turn gave way to low levels of control up to the present.

These findings have implications for control policies and the relevance of a climate-based early-warning system to control itself. Control in these regions is explicitly reactive in the sense that cases in the previous year determine the allocation and planning of resources for insecticide spraying in the next season within the districts. Even in the absence of such policy, the implementation and planning of control in epidemic regions is likely to be implicitly 'reactive'. This is because a feedback is likely to emerge from past disease levels to future human and economic resources allocated to reducing vector abundance and malaria transmission. Whether or not an explicit reactive control policy exists, this feedback can operate at time scales longer than one year, depending in a complex way on the perception of the problem and availability of resources. Thus, our findings have implications beyond the specific district of Kheda to other regions of epidemic malaria where irrigation and its concomitant development underlie more effective control measures. They suggest that inter-annual variability in control levels can set the stage for temporal windows of high susceptibility to anomalous weather conditions. They also indicate that remote sensing in indicator regions (such as the Thar desert) can be used to forecast the potential risk of an outbreak given regional climate variability, especially when control levels fall to low levels.

The dynamical model of mosquito abundance in an irrigated landscape further illustrates the persistent role of climate forcing for disease risk under irrigation. Under irrigation, the seasonal relationship between rainfall and mosquitoes is altered, but only in the non-epidemic months (of low rainfall). In the epidemic months, mosquito abundance continues to respond to rainfall events and, therefore, the inter-annual variability of mosquito abundance after the rainfall season remains unaltered (See Additional file 11, Figure 11, Panel D). This is consistent with the scenario of no decrease in the risk of large epidemics in Kheda with increased irrigation, and the persistence of climatic variability as an important factor, now modulated by levels of control.

Entomological studies may also shed light on this conclusion. *Anopheles culicifacies*, the principal malaria vector in rural areas of India, is mainly present in riverine and canal areas, where two peaks in vector abundance are observed yearly, one in the monsoon season (Jul-Aug) and another in March-April associated with irrigated rice fields [25]. Although temperatures in March increase enough to support mosquito development, temperatures become very high in May-June, leading to the decline of vectors' abundance and lifespan. The short vector longevity during winter leads to a low sporogony rate, and therefore, a low transmission rate for this period. In the monsoon season, however, optimum temperatures and humidity, and extensive areas for breeding, generate suitable conditions for parasite development and large vector populations.

Finally, this study has considered the particular scale of the district, at which the epidemiological data were aggregated. This may not match well the scales at which irrigation influences socioeconomic conditions. In this regard, the complexity of the interaction between malaria dynamics and land-use change, and the long-term consequences of the interaction between socio-economic determinants and malaria dynamics, deserve a closer examination. At local scales, irrigation can be associated with high mosquito populations, but not necessarily with high incidence, a phenomenon known as the paddies paradox [26,27]. Irrigation also may provide new ways to enhance wealth that subsequently improve education levels, housing conditions, or other types of protective measures taken by the individuals in a community or population [28,29]. Therefore, in addition to control measures implemented at larger spatial scales, the observed decoupling between climate variability and malaria can also reflect long-term changes in socio-economic drivers such as better coverage by health facilities, self protective measures, and house improvements. Regardless of the specific mechanism, in areas where the risk itself persists for anomalous climatic conditions, it would be beneficial to incorporate predictions of this risk based on remote sensing tools in the planning of spray interventions.

Further work is needed to understand the connection between agriculture, mosquitoes, human behaviour, and wealth in human-modified landscapes at different spatial and temporal scales. At the large scale of districts, these findings underscore the differential effects of irrigated landscapes on malaria's risk and predictability, including the possibility of unexpected epidemics that are more difficult to predict because of the complex interaction between climate forcing and control efforts. At lower scales, to understand the role of remote sensing in predicting epidemic risk in different agricultural landscapes and how this risk changes in space and time, more research is clearly needed.

## Conclusion

Remote sensing (the vegetation index known as NDVI) provides a useful predictor of malaria epidemics in regions with low levels of irrigation. Increased irrigation modifies the coupling between climatic forcing and malaria's inter-annual variability. This decoupling appears to reflect the effect of control measures rather than a reduction in disease risk. Thus, early-warning systems based on remote sensing in regional indicator regions remain of value to control itself and to the preparedness for public health responses. In addition, reactive control policies may lead to unexpected large epidemics in areas with increased irrigation, when anomalous rainfall coincides with relaxations of control. Prediction efforts coupled to non-reactive control would be of particular value in the transition stage from largely rainfall-driven epidemics to a more permanent reduction of the malaria risk that would accompany socio-economic development and increased irrigation.

## Competing interests

The authors declare that they have no competing interests.

## Authors' contributions

AB, MP, MJB and RCD conceived of the study. AB conducted the statistical analyses. AB and AD built and analysed the mosquito-irrigation model. AB, MP, MJB and AD drafted the manuscript. RD and HS participated in the data collection and provided input on both the draft and malaria in the region, including public health intervention policies. All authors read and approved the final manuscript.

## Supplementary Material

Additional file 1 **Time series of malaria incidence**. The y-axis represents the monthly number of cases per 100,000 people. Note that the range in the y-axis varies across districts. For comparison purposes, see Additional file 2: Figure S2. Click here for file

Additional file 2 **Box-plots of malaria incidence and NDVI**. The first row shows the average and the range of anomalies of cases (in logarithmic scale) for each district in a gradient of irrigation intensity. The second row shows NDVI from the time series inside the districts. Click here for file

Additional file 3 **A simple model of mosquito population dynamics, rainfall and irrigation**. Click here for file

Additional file 4 **Graphical representation of the model**. The land inside the district is divided into irrigated and non-irrigated agriculture (*i *and *n*) and into other uses (*p*). A network of canals drains the water that precipitates on *p *to supply the production of irrigated agriculture. Click here for file

Additional file 5 **Coefficient of variation and seasonality of NDVI**. BMP and Kheda both exhibit low coefficients of variation (red colours) and a peak in NDVI in the month of January. (In the left side of both figures, the red coloured areas delineate the irrigation tract associated with the Indus River in neighbouring Pakistan.) Click here for file

Additional file 6 **Correlation maps using MODIS images**. Spearman rank correlation between September NDVI from MODIS and malaria incidence for a specific district in the epidemic season (the sum of the cases for October, November and December). Note that the dataset consists of ten years (and only 7 years for Barmer). At a significant level of 0.1, evidence for an association between malaria and NDVI at the regional level is present for both Barmer and Kutch. This pattern is less pronounced, however, than for the NOAA NDVI data because of the shorter length of the time series for MODIS NDVI. Click here for file

Additional file 7 **Wavelets analysis (continuation)**. Similar to Figure 4, but for the three districts in the middle part of the irrigation gradient. The picture shows that as irrigation intensified, the 1 year signal became stronger over longer periods of time, both for incidence (Panel A) and NDVI (Panel B). Click here for file

Additional file 8 **Total rainfall, insecticide application and number of cases recorded in Kheda**. The amount of insecticide use corresponds to the proportion of the state population covered by spray activity in that particular year. Rainfall and cases are the total values for the year. Note that in 2004 the large number of cases coincides with anomalous conditions of rainfall and a relatively low level of insecticide application, not just that year but also for a number of previous years. In 1997 and 2001 similar anomalous rainfall conditions did not produce this large number of cases. (Insecticide use data were missing for 2003; the value in the table was predicted by a linear regression of insecticide use at the district level as a function of insecticide use for 10 talukas, administrative units within the district; regression coefficient 0.979). No rainfall data after 2006 are available at this point. Click here for file

Additional file 9 **Malaria predictability based on NDVI v/s rainfall**. The x-axis shows the month of the year used to fit a linear model of the number of cases in the epidemic season (October to December). The y-axis shows the corresponding R-squared value. NDVI is a better predictor than rainfall one month prior (September; dashed line) to the epidemic season (October-November-December) for Barmer, Bikaner and Kutch. For BMP, rainfall from Banaskantha is a better predictor. For Kheda, neither NDVI, nor rainfall, are good predictors of epidemics. Click here for file

Additional file 10 **Maximum, minimum and yearly average mosquito abundance**. Panel A shows that the minimum mosquito abundance increases as the total area under irrigation increases, however its maximum does not change. Panel B shows that mosquito abundance increases linearly with the proportion of land under irrigation (*i*). Click here for file

Additional file 11 **Seasonal and inter-annual correlation**. Correlation between mosquito (M) and precipitation (P) with non-irrigated agriculture (*i *= 0; panel, A and C), and with 30 percent of the landscape under irrigated agriculture (*i *= 0.3; panels B and D). Seasonal correlation in panels A and B and inter-annual correlation in panels C and D. The values for the rest of the parameters are: *n *= 0.1; *p *= (1 *- n - i*); *e *= 30; *d *= 200; *c *= 0.1; *b *= 120; *μ *= 18; *ρ *= 0.8; *ω *= 0.1; *f_n _*= *f_i _*= 3; *r_1 _*= 200; *r_0 _*= 0.99; *m_1 _*= 200; *m_0 _*= 0.99; *h *= 5; *α_n _*= 2; *α_i _*= 3. The annual cycle leads to the change in correlation, but not the actual inter-annual variability. Click here for file
