# Supplementary figures and images for: Climate forcing and desert malaria: the effect of irrigation
Source: Malar J. 2011 Jul 14;10:190. doi: 10.1186/1475-2875-10-190 (PMC3155970; doi:10.1186/1475-2875-10-190)

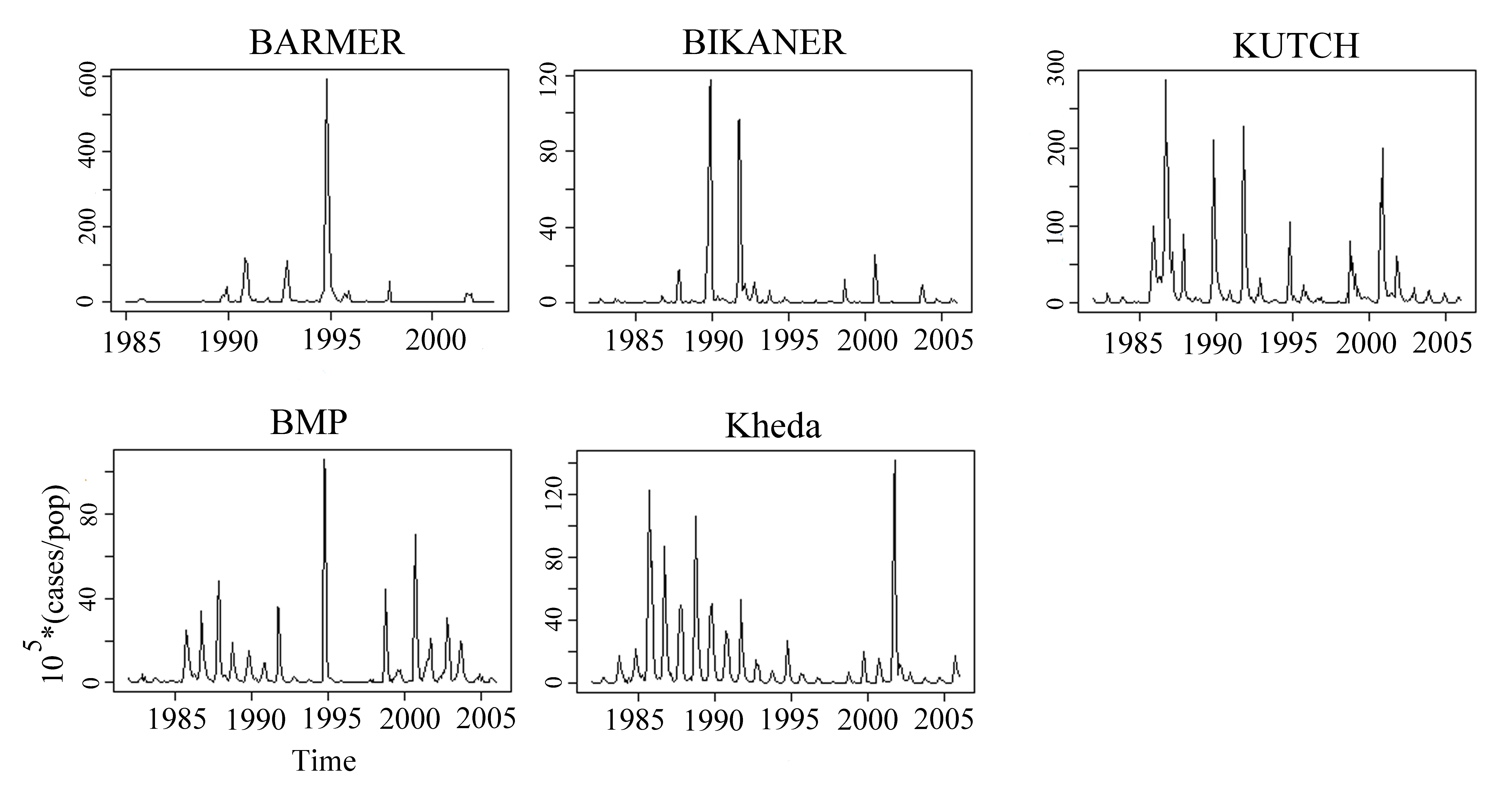

Supplement: Additional file 1 — Time series of malaria incidence. The y-axis represents the monthly number of cases per 100,000 people. Note that the range in the y-axis varies across districts. For comparison purposes, see Additional file 2: Figure S2. [file 1475-2875-10-190-S1.TIFF]

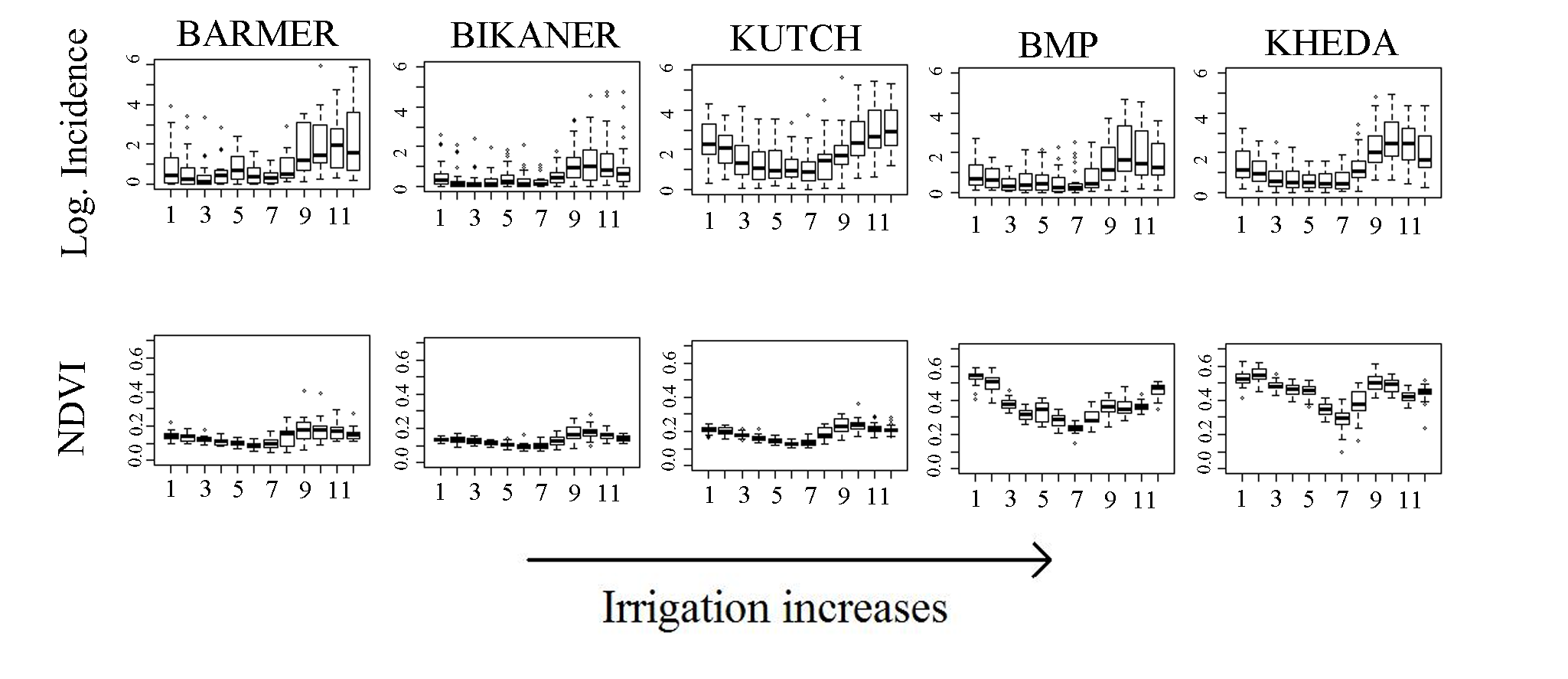

Supplement: Additional file 2 — Box-plots of malaria incidence and NDVI. The first row shows the average and the range of anomalies of cases (in logarithmic scale) for each district in a gradient of irrigation intensity. The second row shows NDVI from the time series inside the districts. [file 1475-2875-10-190-S2.TIFF]

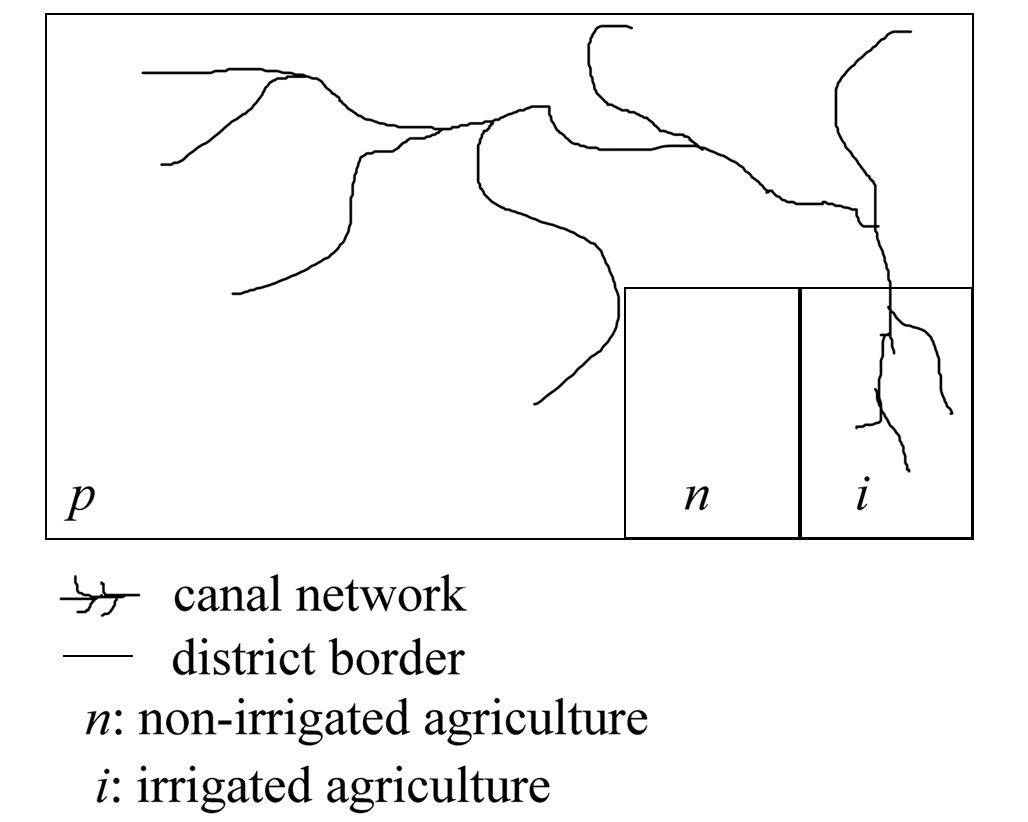

Supplement: Additional file 4 — Graphical representation of the model. The land inside the district is divided into irrigated and non-irrigated agriculture (i and n) and into other uses (p). A network of canals drains the water that precipitates on p to supply the production of irrigated agriculture. [file 1475-2875-10-190-S4.TIFF]

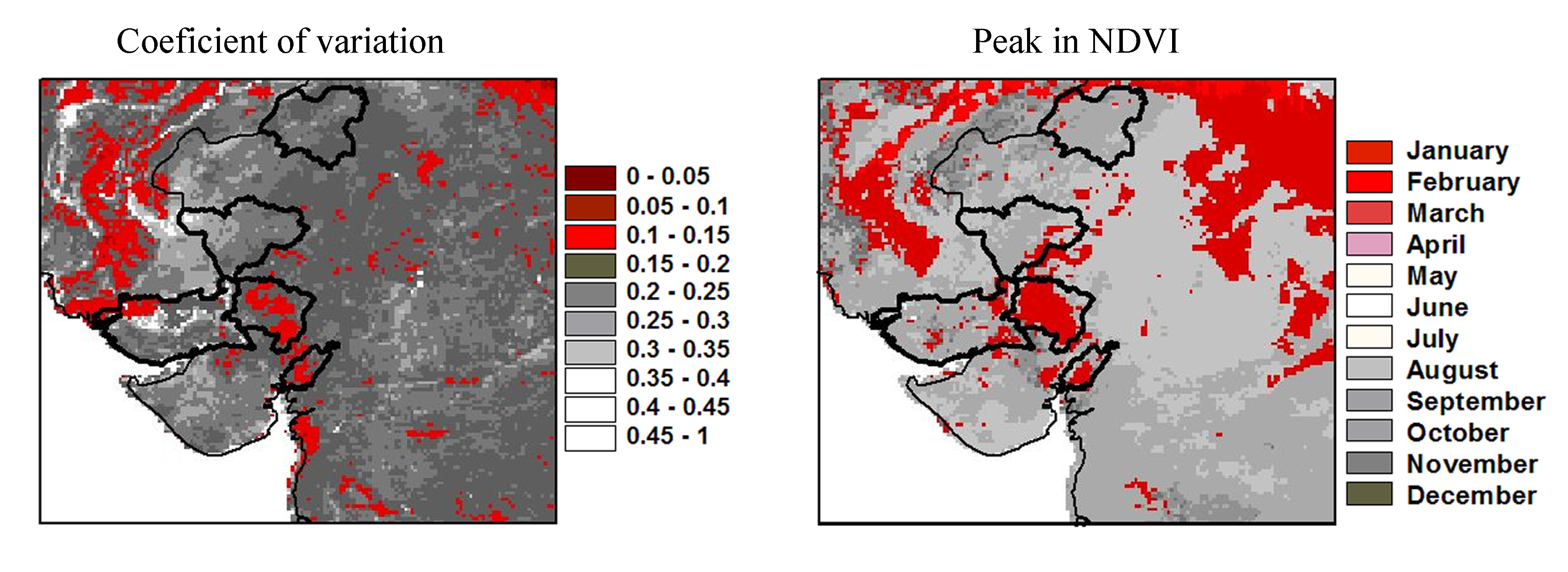

Supplement: Additional file 5 — Coefficient of variation and seasonality of NDVI. BMP and Kheda both exhibit low coefficients of variation (red colours) and a peak in NDVI in the month of January. (In the left side of both figures, the red coloured areas delineate the irrigation tract associated with the Indus River in neighbouring Pakistan.) [file 1475-2875-10-190-S5.TIFF]

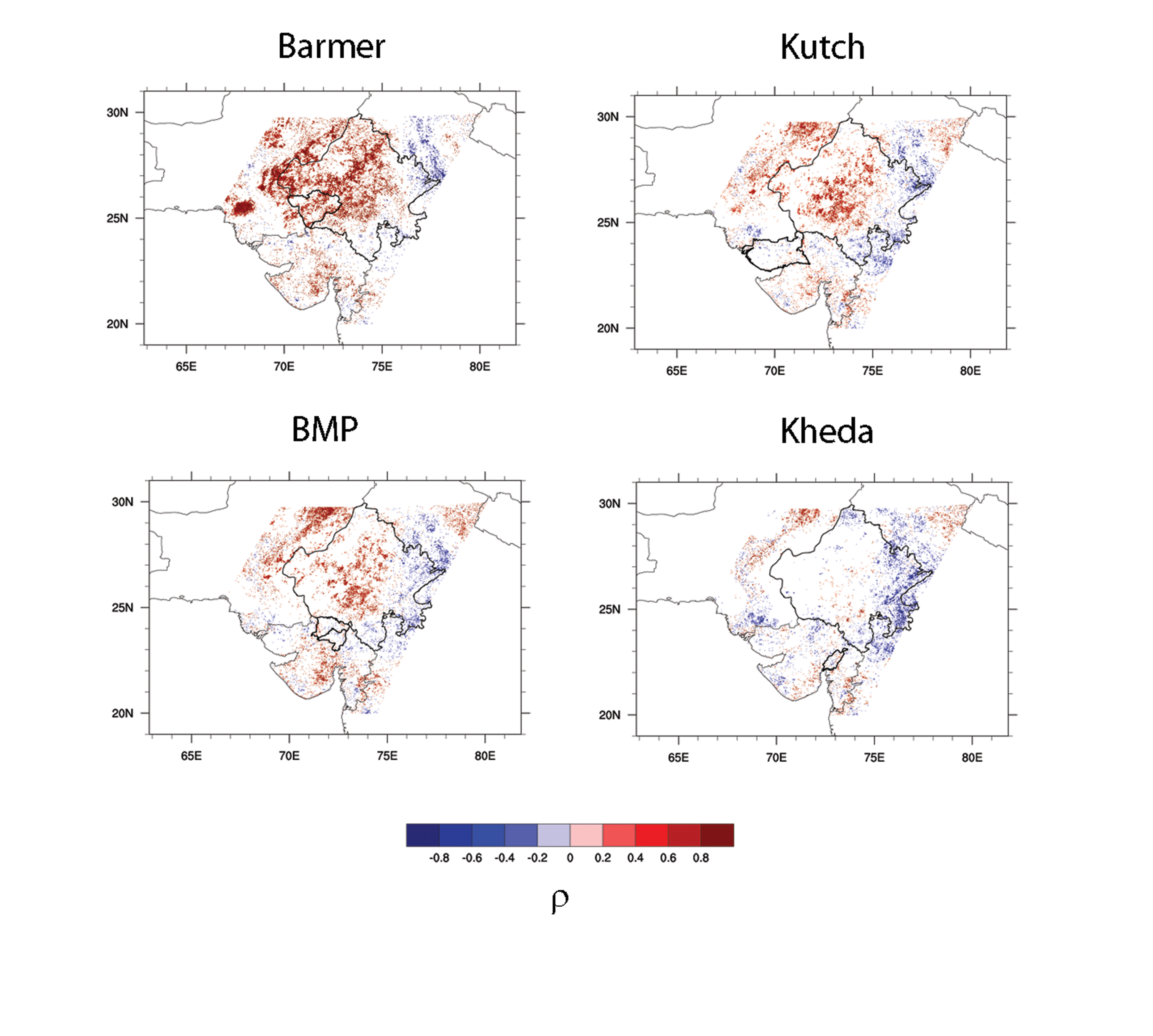

Supplement: Additional file 6 — Correlation maps using MODIS images. Spearman rank correlation between September NDVI from MODIS and malaria incidence for a specific district in the epidemic season (the sum of the cases for October, November and December). Note that the dataset consists of ten years (and only 7 years for Barmer). At a significant level of 0.1, evidence for an association between malaria and NDVI at the regional level is present for both Barmer and Kutch. This pattern is less pronounced, however, than for the NOAA NDVI data because of the shorter length of the time series for MODIS NDVI. [file 1475-2875-10-190-S6.TIFF]

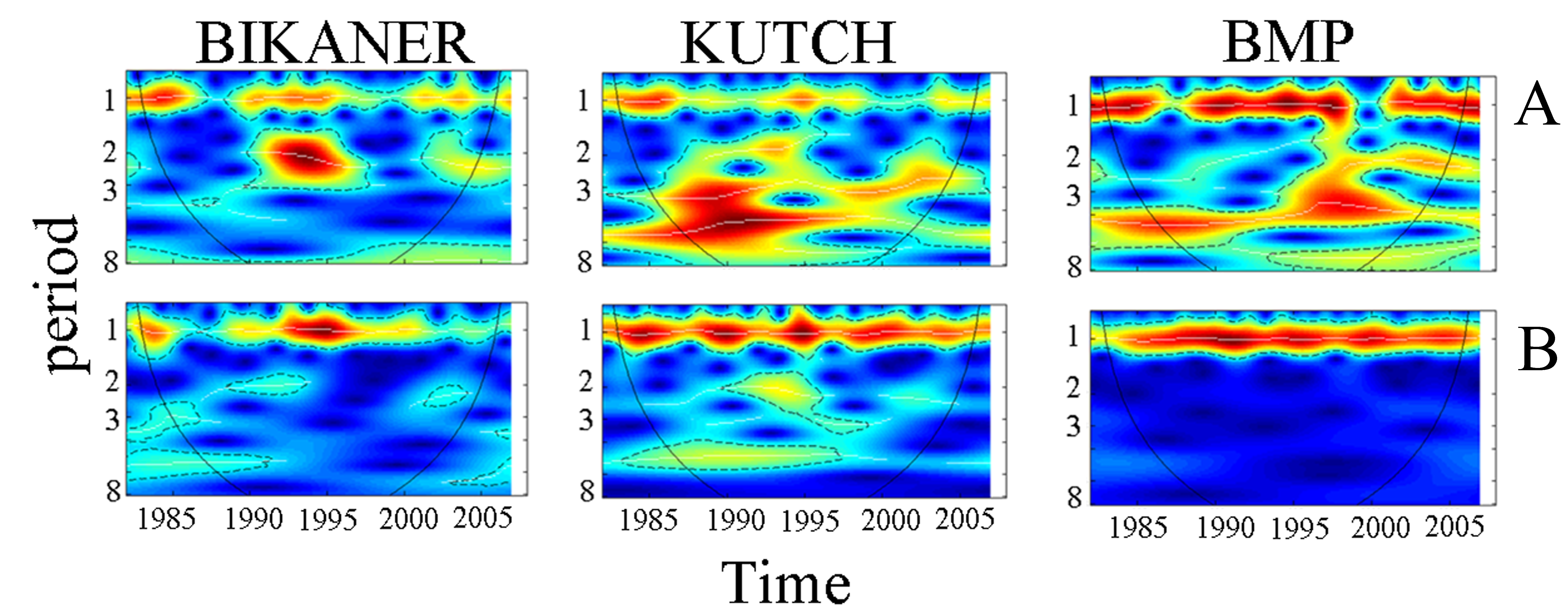

Supplement: Additional file 7 — Wavelets analysis (continuation). Similar to Figure 4, but for the three districts in the middle part of the irrigation gradient. The picture shows that as irrigation intensified, the 1 year signal became stronger over longer periods of time, both for incidence (Panel A) and NDVI (Panel B). [file 1475-2875-10-190-S7.TIFF]

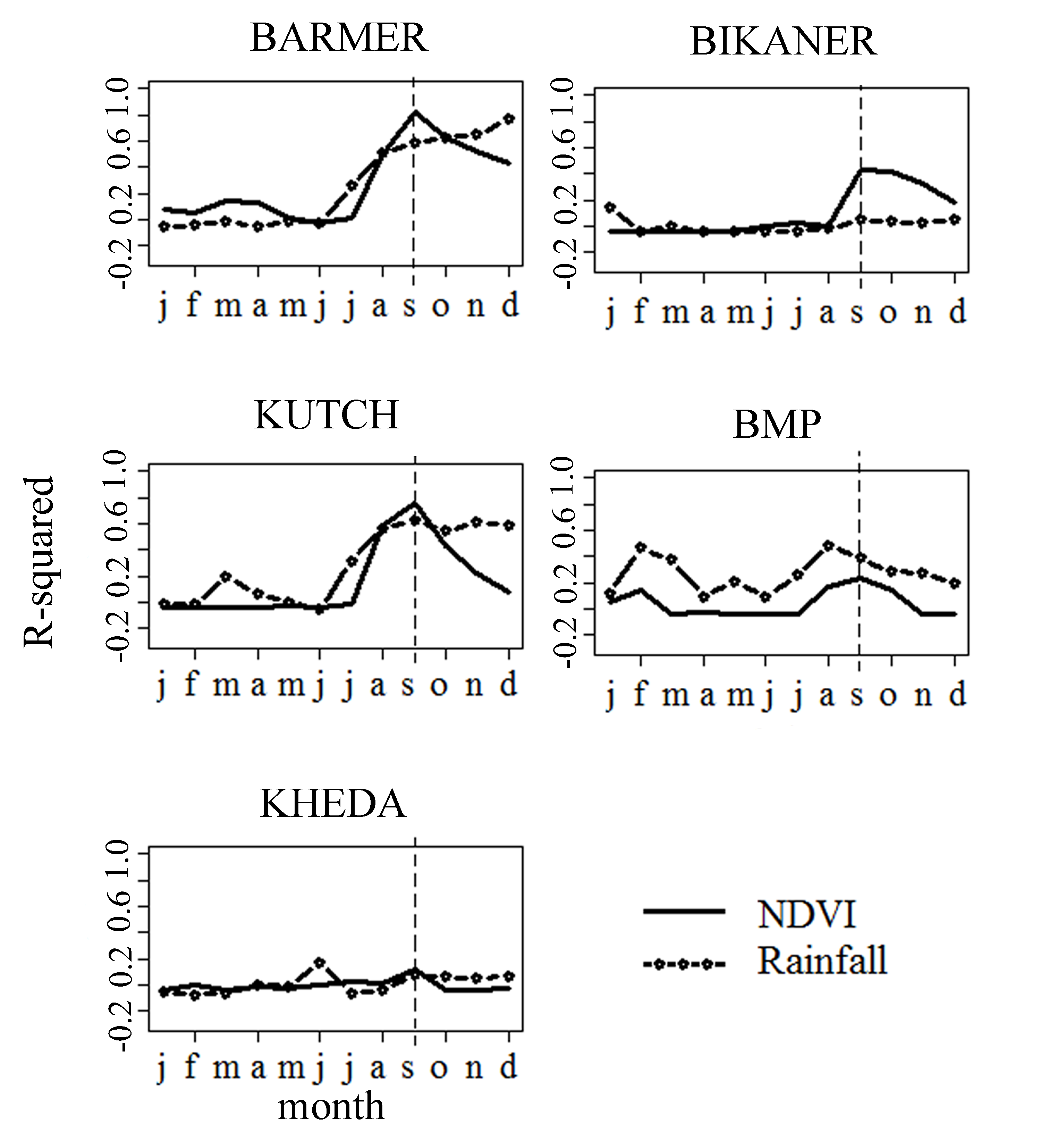

Supplement: Additional file 9 — Malaria predictability based on NDVI v/s rainfall. The x-axis shows the month of the year used to fit a linear model of the number of cases in the epidemic season (October to December). The y-axis shows the corresponding R-squared value. NDVI is a better predictor than rainfall one month prior (September; dashed line) to the epidemic season (October-November-December) for Barmer, Bikaner and Kutch. For BMP, rainfall from Banaskantha is a better predictor. For Kheda, neither NDVI, nor rainfall, are good predictors of epidemics. [file 1475-2875-10-190-S9.TIFF]

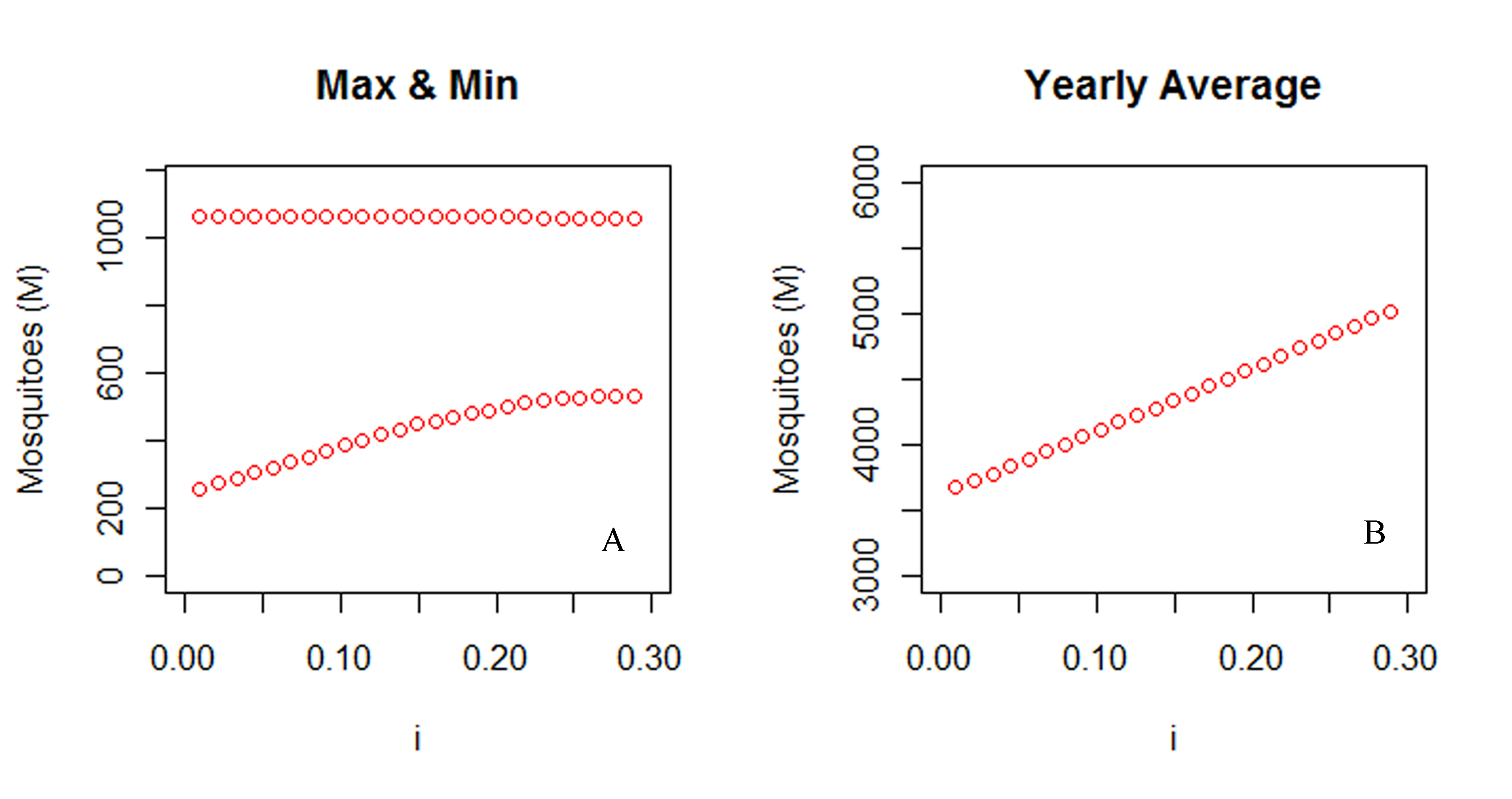

Supplement: Additional file 10 — Maximum, minimum and yearly average mosquito abundance. Panel A shows that the minimum mosquito abundance increases as the total area under irrigation increases, however its maximum does not change. Panel B shows that mosquito abundance increases linearly with the proportion of land under irrigation (i). [file 1475-2875-10-190-S10.TIFF]

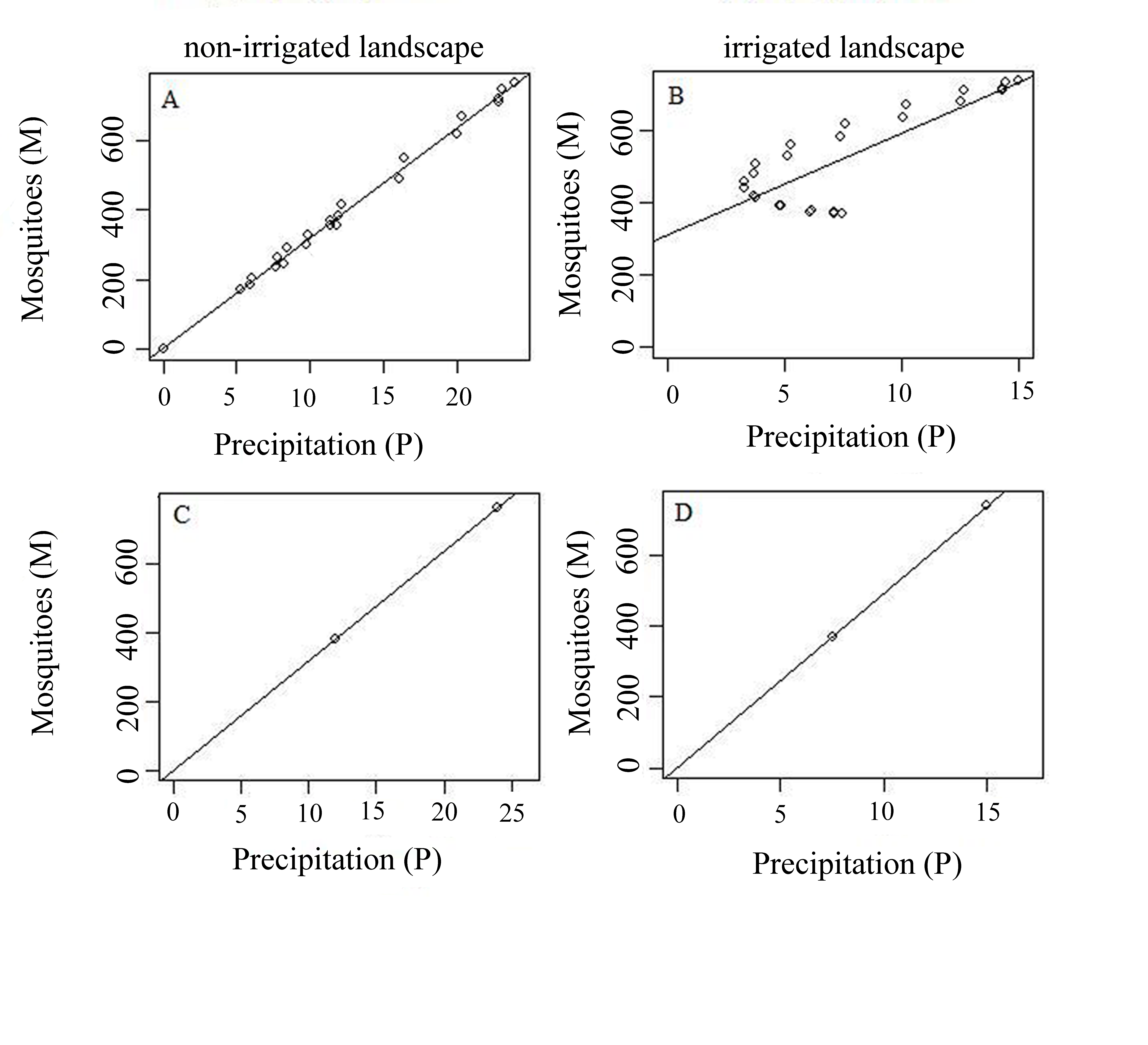

Supplement: Additional file 11 — Seasonal and inter-annual correlation. Correlation between mosquito (M) and precipitation (P) with non-irrigated agriculture (i = 0; panel, A and C), and with 30 percent of the landscape under irrigated agriculture (i = 0.3; panels B and D). Seasonal correlation in panels A and B and inter-annual correlation in panels C and D. The values for the rest of the parameters are: n = 0.1; p = (1 - n - i); e = 30; d = 200; c = 0.1; b = 120; μ = 18; ρ = 0.8; ω = 0.1; fn = fi = 3; r1 = 200; r0 = 0.99; m1 = 200; m0 = 0.99; h = 5; αn = 2; αi = 3. The annual cycle leads to the change in correlation, but not the actual inter-annual variability. [file 1475-2875-10-190-S11.TIFF]
