# Supplementary material for: Climate forcing and desert malaria: the effect of irrigation
Source: Malar J. 2011 Jul 14;10:190. doi: 10.1186/1475-2875-10-190 (PMC3155970; doi:10.1186/1475-2875-10-190)
Supplement: Additional file 3 — A simple model of mosquito population dynamics, rainfall and irrigation. [file 1475-2875-10-190-S3.PDF]

A simple model of mosquito population dynamics, rainfall and irrigation

A relatively straightforward model can be constructed that examines the underlying dynamic interactions between rainfall, irrigated agriculture, and mosquito abundance. We use this model to interpret and illustrate some of the patterns observed in the statistical analysis of the empirical data.

In order to keep the model simple, we consider the dynamics of the mosquito population in a landscape composed of agricultural and non agricultural land (See Additional file 4: Figure S4). Specifically, we define D the total proportions of a district's land as follows:

$D = 1 = a + p$.

where a is the proportion of the total area that is designated for agriculture and p is the proportion designated for other uses. We also differentiate between seasonal agriculture and agriculture under irrigation, such that

$a = i + n$

where i and n are the proportions of land covered by irrigated and non-irrigated agriculture, respectively.

Five equations describe the system:

$$\frac{dP}{dt} = p * f(\pi) - e * P - \frac{d}{e} * P * i$$

$$\frac{dW}{dt} = \left(\frac{d}{e} * P - c * e * W - f(\psi) \right) * i$$

$$\frac{dA_n}{dt} = f(\pi) * y_n * n - h * A_n$$

$$\frac{dA_i}{dt} = f(\psi) * y_i * i - h * A_i$$

$$\frac{dM}{dt} = b * \left(\rho * P + \alpha_n * A_n + \alpha_i * A_i + \omega * W \right) - \mu * M$$

P is the total volume of water that stands in p and W is the total volume of water stored in the canal network. A_n and A_i denote the total agriculture yield obtained in *a (respectively, irrigated and non-irrigated),* and M is the mosquito abundance.

Here f(π) is the bi-annual rain fall cycle, which we characterize using

$r_1*(1 + r_0*\sin(2\pi f_r)) + m_1*(1 + m_0*\sin(2\pi f_m))$. m_1 is the average extra water due to monsoon events with frequency f_m = 1/24 months, and r_1 is the average annual rainfall with f_r = 1/12. e is the evaporation rate of water in puddles, d is the rate at which water is drained into W, c is the relative rate at which irrigation evaporates (we assume a value around 0.1), and $f(\psi)$ is the rate at which water storage in W is supplied to

irrigated agriculture. $f(\psi)$ is also a sin wave function with a period of one year and a maximal peak lagging 6 months after that of the rainfall season. y_n and y_i are the conversion constants from water to seasonal and irrigated crop production, and h is the crop harvesting rate. Although crop yield most likely differs for irrigated versus non-irrigated systems, and this may influence mosquito breeding preference, we set $y_n = y_i$ to focus on the large scale effect of increasing the area designated for irrigated agriculture. $\omega*W$ and $\rho*P$ correspond to the contribution of the canal network and accumulated water on p to mosquito abundance, respectively, and $\alpha_n A_n$ and $\alpha_i A_i$ correspond to the contribution of non-irrigated (seasonal) and irrigated agriculture. In our simulations we assume that $\alpha_n < \alpha_n$.

The total yield of non-irrigated agriculture is assumed to be proportional to the amount of rain that falls directly in n. This is denoted by $f(\pi)*n$, where $f(\pi)$ is the total rain that falls onto D.

Mosquito birth rate is assumed to be entirely dependent upon available water in the district (agriculture, non-agriculture land, and canal network), but the potential for mosquitoes to breed varies depending on land-use. We ignore larval stages and associated time delays and simply assume that mosquitoes die at a constant rate μ. It would be relatively straightforward to add additional stages for mosquito biting rates and the abundance of infected and susceptible hosts, but the details of this would obscure the main points we wish to make, so we simply assume that mosquito abundance is a good index of transmission potential for malaria.

We initially used the model to examine the seasonal (annual) and inter-annual correlations between rainfall (measured by P) and mosquito abundance M. Additional file 11: Figure S11 illustrates the correlations with and without irrigation: With no irrigation, there is a strong relationship between seasonal rainfall and mosquito abundance (panel A). When we increase the area of irrigated agriculture, then we see a weaker correlation between rainfall and mosquito abundance (Additional file 11: panel B). However, irrigation does not affect the inter-annual correlation, as illustrated in panels C and D.

We also examine the long-term consequences for epidemic risk of increasing the area designated for agriculture under irrigation. We simulated the model for a range from almost no irrigated land $i = 0.01$, to thirty percent irrigated landscape, and measure the total mosquito abundance in a year and the maximal and minimal values (See Additional file 10: Figure S10, panels A and B). These simulations show that irrigation leads to an increase in the mosquito abundance (panel B), but it does not affect the maximum, which is still controlled by the amount of rainfall (panel A).
